# Establishment and comparison of *in situ* detection models for foodborne pathogen contamination on mutton based on SWIR-HSI

**DOI:** 10.3389/fnut.2024.1325934

**Published:** 2024-02-09

**Authors:** Zongxiu Bai, Dongdong Du, Rongguang Zhu, Fukang Xing, Chenyi Yang, Jiufu Yan, Yixin Zhang, Lichao Kang

**Affiliations:** ^1^College of Mechanical and Electrical Engineering, Shihezi University, Shihezi, China; ^2^Analysis and Test Center, Xinjiang Academy of Agricultural and Reclamation Science, Shihezi, China; ^3^Key Laboratory of Northwest Agricultural Equipment, Ministry of Agriculture and Rural Affairs, Shihezi University, Shihezi, China; ^4^Engineering Research Center for Production Mechanization of Oasis Characteristic Cash Crop, Ministry of Education, Shihezi University, Shihezi, China

**Keywords:** hyperspectral imaging, deep learning, machine learning, foodborne pathogens, mutton

## Abstract

**Introduction:**

Rapid and accurate detection of food-borne pathogens on mutton is of great significance to ensure the safety of mutton and its products and the health of consumers.

**Objectives:**

The feasibility of short-wave infrared hyperspectral imaging (SWIR-HSI) in detecting the contamination status and species of *Escherichia coli* (EC), *Staphylococcus aureus* (SA) and *Salmonella typhimurium* (ST) contaminated on mutton was explored.

**Materials and methods:**

The hyperspectral images of uncontaminated and contaminated mutton samples with different concentrations (10^8^, 10^7^, 10^6^, 10^5^, 10^4^, 10^3^ and 10^2^ CFU/mL) of EC, SA and ST were acquired. The one dimensional convolutional neural network (1D-CNN) model was constructed and the influence of structure hyperparameters on the model was explored. The effects of different spectral preprocessing methods on partial least squares-discriminant analysis (PLS-DA), support vector machine (SVM) and 1D-CNN models were discussed. In addition, the feasibility of using the characteristic wavelength to establish simplified models was explored.

**Results and discussion:**

The best full band model was the 1D-CNN model with the convolution kernels number of (64, 16) and the activation function of tanh established by the original spectra, and its accuracy of training set, test set and external validation set were 100.00, 92.86 and 97.62%, respectively. The optimal simplified model was genetic algorithm optimization support vector machine (GA-SVM). For discriminating the pathogen species, the accuracies of SVM models established by full band spectra preprocessed by 2D and all 1D-CNN models with the convolution kernel number of (32, 16) and the activation function of tanh were 100.00%. In addition, the accuracies of all simplified models were 100.00% except for the 1D-CNN models. Considering the complexity of features and model calculation, the 1D-CNN models established by original spectra were the optimal models for pathogenic bacteria contamination status and species. The simplified models provide basis for developing multispectral detection instruments.

**Conclusion:**

The results proved that SWIR-HSI combined with machine learning and deep learning could accurately detect the foodborne pathogen contamination on mutton, and the performance of deep learning models were better than that of machine learning. This study can promote the application of HSI technology in the detection of foodborne pathogens on meat.

## 1 Introduction

Mutton is one of the most popular meats in the world due to its unique flavor and rich protein content (1). Because of the rich nutrients, it is a good substrate for the growth and reproduction of various microorganisms. Pathogenic bacteria are important factors that affect food safety and threaten human health. The current Chinese national standard (GB 29921–2013) for Food Safety-Limits of Pathogenic bacteria in Food stipulates the indicators, sampling schemes, limits and detection methods of pathogenic bacteria in different categories of food (2). Among them, the common pathogens in raw meat include *Escherichia coli* (EC), *Staphylococcus aureus* (SA) and *Salmonella typhimurium* (ST), etc. It is required that EC and ST could not be detected in 25 g of raw meat and that the SA content should not exceed 1,000 CFU/g. Consuming foods contaminated with these bacteria can cause vomiting, diarrhea or even death (3). Therefore, rapid, sensitive and specific detection of foodborne pathogens has become an urgent requirement in health safety, food quality control and other aspects.

The two current standards used to detect bacterial foodborne pathogens are conventional culture and polymerase chain reaction methods (4). However, these methods have some problems such as environmental pollution, time consuming, and strict requirements for operators and operating environments. Optical rapid detection technology can use the unique optical characteristics of microbial samples to identify them, which opens up new avenues for microbial research. Due to their fast, green and easy operation, they have shown good application prospects in the detection of pathogenic bacteria in recent years (5–7). Among them, hyperspectral imaging (HSI) technology can simultaneously collect spatial, spectral and radiation information of the object to be measured, and has become one of the most powerful technologies for food quality and safety detection (8). Currently, studies on the use of HSI to detect meat freshness (9), adulteration (10, 11), the total number of colonies (12) and parasites (13) have been widely reported. There are also many reports on the use of HSI combined with machine learning for the detection of foodborne pathogens. Kammies et al. (14) used HSI combined with PLS-DA to effectively distinguish gram-positive and negative bacteria such as EC, SA, and ST on agar plates. Feng et al. (15) used HSI and multi-spectral imaging combined with invasive weed optimization (IWO) to classify EC, *Listeria monocytogenes*, and SA on agar plates. Bonah et al. (16) used HSI to classify foodborne pathogens growing on agar plates, and combined with partial least squares regression (PLSR) algorithm to rapidly detect the content of EC and SA in fresh pork, and realized visual detection. Gu et al. (17) combined the HSI technique with commonly used optimization algorithms to classify EC, SA, and ST on bacteriolytic broth, plate counting agar, and tryptone soy agar. Unger et al. (18) developed and evaluated a low-cost HSI system to identify single and mixed foodborne pathogen strains in dairy products. The above studies were all based on the spectra of pathogenic bacteria colonies on the culture medium. If the pathogenic bacteria on the meat can be detected *in situ*, the process of isolation and culture can be avoided, so as to shorten the detection time and save the detection cost. However, only a few studies have examined *in situ* pathogenic bacteria of meat. Bonah et al. (19) used visible near infrared (Vis–NIR) HSI and partial least squares regression (PLSR) algorithm to rapidly monitor the content of foodborne pathogens (EC and SA) in fresh pork longissimus muscle. Qiu et al. (4) explored the feasibility of using hyperspectral imaging to establish an efficient classification model for qualitative detection of SA in chicken breast meat. When *in situ* detection of pathogenic bacteria in chicken and pork was carried out in those studies, only the content prediction and concentration division of a single strain were considered. In practice, it is not certain whether mutton is contaminated with pathogenic bacteria and the species of pathogenic bacteria. Therefore, it is very important to detect the contamination status of pathogenic bacteria in mutton and identify the species of pathogenic bacteria to ensure the safety of mutton and its products.

At present, the processing of hyperspectral data is mainly using traditional machine learning methods for preprocessing, feature wavelength extraction and modeling. With the development of computer technology and artificial intelligence, deep learning methods have been developed rapidly. It can automatically extract and continuously optimize features by pre-training the model, and can quickly process a large number of data. Better performance and higher accuracy make it show superior performance in spectral data processing, and it has been widely used in spectral data processing (20). Among them, convolutional neural network (CNN) is a typical feed-forward neural network, which can automatically extract deep features from the original data through convolution and pooling structure (10, 21). In the aspect of spectral data processing, there are numerous researches using one-dimensional convolutional neural network (1D-CNN) (22–26). At present, the application of deep learning in the field of rapid detection of pathogenic bacteria is mainly based on the microscopic scale, and there are few studies on the macroscopic scale. Kang et al. (27–30) used hyperspectral microscopic imaging (HMI) combined with various advanced deep learning frameworks such as long short term memory (LSTM) network, deep residual network (ResNet) and 1D-CNN to do in-depth research on pathogenic bacteria at the microscopic scale, and realized the rapid classification of foodborne pathogenic bacteria at the cellular level. An artificial intelligence assisted HMI method was developed that directly processes the spectra of five common foodborne pathogens from different rois. In addition, Tao et al. (31) combined HMI technology and Buffer Net deep learning algorithm to construct an AI assisted system for automatic and rapid bacterial genus discrimination, which can identify pathogenic bacteria at the single-cell level with high accuracy in a cheap, fast, and automated manner. In addition, the existing hyperspectral band in the field of pathogen detection is mostly 400–1,000 nm (VNIR-HSI). The reflectance in the band range of VNIR-HSI is greatly affected by color. Since mutton is red, the influence of its own color on the spectra is large in this band range. The contamination of mutton by pathogenic bacteria mainly causes changes in the chemical composition of mutton. The spectra of SWIR-HSI at 1000–2500 nm are more suitable for chemical analysis and composition detection of substances. It can reduce the errors caused by mutton itself. And *in situ* detection of pathogenic bacteria in meat using SWIR-HSI has not been re-ported so far.

Therefore, the SWIR-HSI was used to detect the presence of pathogenic bacteria and discriminate the different species of EC, SA and ST on mutton *in situ*. The specific work is as follows: (1) hyperspectral images of uncontaminated mutton samples and samples contaminated with different concentrations of EC, SA and ST were collected, (2) the average spectral information of the region of interest for each sample were extracted for modeling analysis, (3) the 1D-CNN models which were suitable for the detection of pathogenic bacteria contamination status and the discrimination of the pathogenic bacteria species on mutton were constructed and the network structure were optimized by the structural hyperparameters, (4) the influence of spectral preprocessing on different models was explored, (5) the detection models based on characteristic wavelengths were established to explore the feasibility of using machine learning and deep learning algorithms to establish simplified models for the contamination status and species discrimination of foodborne pathogens on mutton. The study explored the feasible of using spectral information combined with machine learning or deep learning algorithms to detect EC, SA and ST contamination status and types on mutton and tried to provide a new idea for the detection of pathogenic bacteria in food.

## 2 Materials and methods

### 2.1 Preparation of bacterial suspension and inoculum

The bacterial strain of SA, ST and EC used in this study was ATCC 25923, ATCC 14028, and ATCC 21520, respectively. All strains were collected from the Microbiology Laboratory of Analysis and Testing Center of Xinjiang Academy of Agriculture and Reclamation Sciences. Nutrient broth medium (Beijing Land Bridge Technology Co., LTD. Beijing, China) was selected as the culture medium, while phosphate buffer solution (PBS) was used to prepare the decimal dilutions at 19.0 g/L and the medium for bacterial counting was prepared by adding bacterial agar powder to the nutrient broth. After autoclaving at 121°C for 15 min and cooling to approximately 50°C, medium making was carried out in a biosafety cabinet. About 15–20 mL of the cooled nutrient broth was poured into the Petri dish and allowed to solidify until set aside. Other materials used in the experiment mainly included phosphate buffer solution (PBS), Petri dishes with a diameter of 90 mm, inoculation rings, pipetting guns and their tips, centrifuge tubes, coating rods and distilled water. The EC, SA and ST stored at −80°C were inoculated into nutrient agar medium and stored in a constant temperature incubator at 37°C for activation and culture for 20 ± 1 h. The total number of colonies was determined according to GB4789.2–2016. The single colonies with appropriate morphology were inoculated in the broth and incubated at 37°C for 4 h. The bacteria solution was diluted 10 times gradient with PBS to obtain 7 different concentrations of bacteria solution (10^8^, 10^7^, 10^6^, 10^5^, 10^4^, 10^3^, 10^2^ CFU/mL). After sealing and packing, the bacteria solution was stored at 4°C for later use.

### 2.2 Sample preparation

The fresh mutton (hind leg part) required for the samples was purchased from Friendship Supermarket (China) in Shihezi City, Xinjiang. After the meat was transported to the laboratory in an incubator, obvious fascia and tissue were removed and divided to make meat samples of about 50 × 30 × 10 mm. The weight of each sample was guaranteed to be 25 ± 1 g. The labeled samples were placed in a disk and sterilized for 30 min in a biosafety chamber using a purple light. Then the hyperspectral image data of 210 uncontaminated samples were collected. Different concentrations of bacteria solution were repeatedly blown to mix well by using a pipette gun, and then 1 mL of bacteria solution was absorbed and evenly spread on the surface of meat. A total of 210 mutton samples contaminated with different types (EC, SA and ST) and concentrations ((10^8^, 10^7^, 10^6^, 10^5^, 10^4^, 10^3^, 10^2^ CFU/mL)) of pathogenic bacteria were collected. Among them, 70 samples were inoculated with EC, SA and ST, respectively, while 10 samples were used for each one of the inoculum levels. The applied samples were left in a biosafety cabinet for 25 min to ensure that the pathogenic bacteria were completely absorbed by the meat. The hyperspectral image data of sample contaminated pathogenic bacteria were collected after ensuring that the water remaining on the sample surface evaporated.

### 2.3 Hyperspectral image acquisition and spectral data extraction

The experiment was carried out at a temperature of 26 ± 1°C and a relative humidity of 30 ± 5%. The line scan push-sweep acquisition system SWIR-HSI mainly includes: Imaging spectrograph (ImSpector N25E 2/3, Spectral Imaging Ltd. Oulu, Finland) with 288 bands in the wavelength range of 1,000–2,500 nm, a CCD camera (Zephir-2.5-320, Photon, Montreal, Canada), light source (six halogen with 150 W), a positioning sample table driven by a stepping motor, a computer equipped with data acquisition software, a dark box, etc. Before sample collection, spectrometer was turned on to preheat for half an hour. Then the operating software of the spectrometer and the loading platform was opened, and the focal length of the hyperspectral camera was adjusted to ensure that the images were clear and not distorted. The motor platform was controlled to move and the hyperspectral images of samples were collected. When samples were collected in this study, the lens height from the sample surface was 25 cm, the displacement stage speed was 70.38 mm/s, and the exposure time was 3.5 ms.

In order to reduce noise and interference factors, the collected sample hyperspectral image data needed to be corrected in black and white. In this study, the original image data (IR) was corrected in black and white by using all-white calibration image data (IW) and all-black calibration image data (IB), and the corrected image data (I) was obtained. The Equation is as follows:

(1) $$I=\frac{I_{R}-I_{B}}{I_{W}-I_{B}}$$

**Figure 1:**
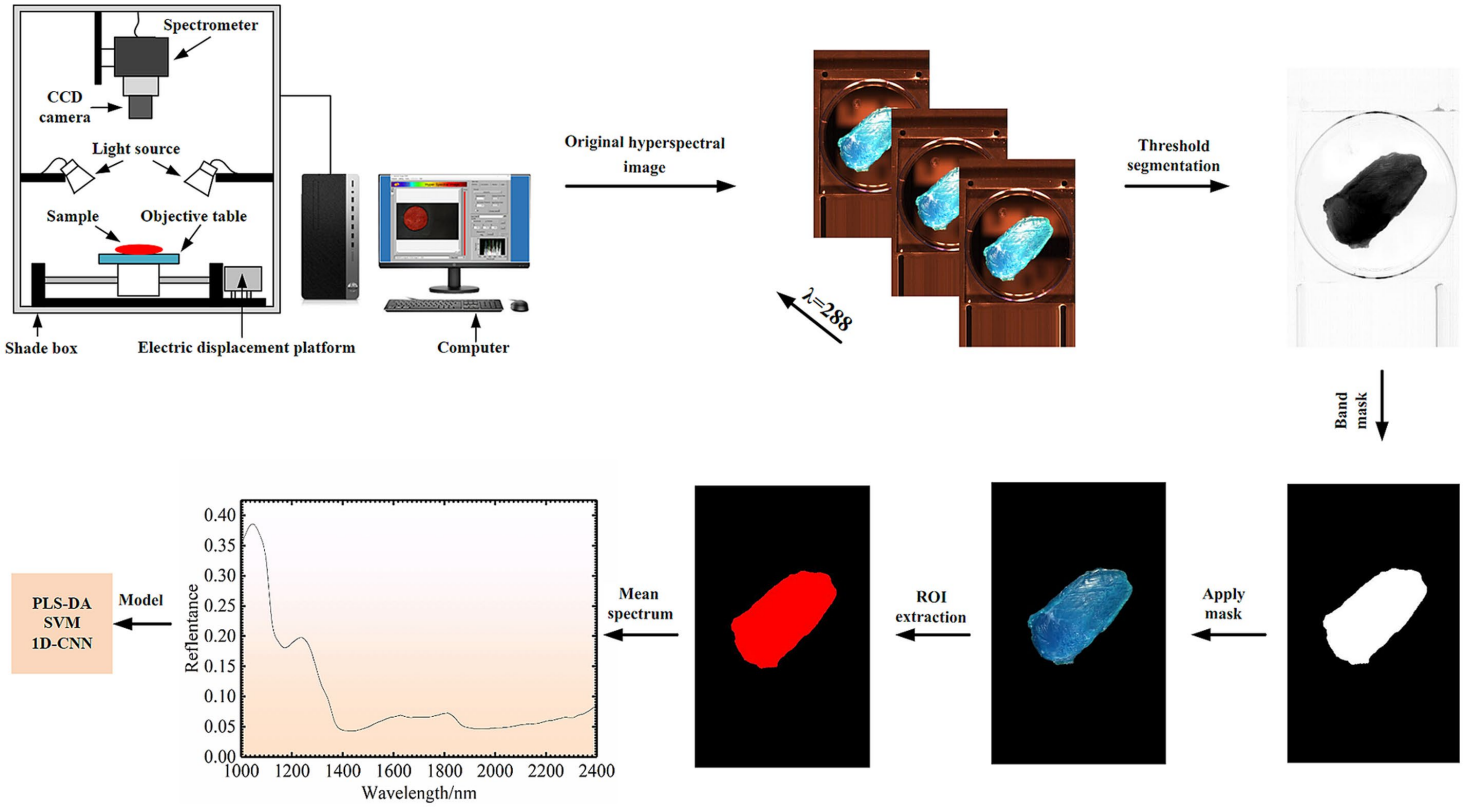
Schematic diagram of the SWIR-HSI acquisition system and data acquisition process.

The whole area of the obtained mutton sample was regarded as the region of interest (ROIs). The ROIs of samples were extracted using band subtraction by ENVI 5.3 spectral processing software (ITT Visual Information Solutions, Boulder, CO, United States). The schematic diagram of the data acquisition system and the spectral data acquisition process is shown in Figure 1.

### 2.4 Preprocessing of spectral information

Since the original spectra of the samples were noisy at wavelengths before 1,000 nm and after 2,400 nm, 223 spectra in the band range of 1,000–2,400 nm were selected for subsequent analysis. When spectral data are obtained, the spectra may be affected by external factors and contain a lot of noise and other interference information. The interference of irrelevant information can be removed by preprocessing the spectra. In this study, the methods of first derivative (1D), second derivative (2D), mean center (MC) and multiplicative scattering correction (MSC) were used to preprocess the original spectral data, and their effects on different models were investigated.

### 2.5 Dataset construction

A total of 210 mutton samples were prepared in this study, and the spectra information of all samples after sterilization was firstly collected as uncontaminated samples. Then 210 mutton samples were contaminated with different types and concentrations of pathogenic bacteria and their HSI data were collected as contaminated samples. A total of 420 mutton samples were used to establish the discrimination model of pathogen contamination status. The 210 contaminated samples were used to establish the discrimination model of pathogen contamination species. When building the model, all data sets were divided into training set, test set and external validation set. Firstly, the training set was used to train the model, and then the model parameters were adjusted according to the test set to obtain a more accurate model. Finally, the stability of the model was verified by the external validation set. When establishing the model, 1 contaminated mutton sample with each concentration of each pathogen was randomly selected as the external validation set, 2 samples were selected as the test set, and the remaining 7 samples were selected as the training set. The spectral data of each sample before uncontaminated were divided into the corresponding dataset. The number of samples in the training set, test set and external validation set for the detection model of pathogen contamination status on mutton was 294, 84 and 42, respectively. The number of samples in the training set, test set and external validation set of species discriminant model for mutton contamination pathogens pathogen was 147, 42 and 21, respectively.

### 2.6 Characteristic wavelengths extraction

Characteristic wavelengths extraction can enhance the explanatory power of the analysis by removing redundant and less informative spectral features. It plays a crucial role in machine learning, but their impact on deep learning is unclear. To explore the feasibility of using four algorithms to establish simplified models for bacterial contamination status and species discrimination, the common characteristic wavelength extraction methods genetic algorithm (GA), competitive adaptive reweighting algorithm (CARS) and successive projections algorithm (SPA) (32, 33) were used to extract the characteristic wavelengths closely related to the bacterial contamination status and the species of pathogenic bacteria, and the simplified models were established based on the characteristic wavelengths.

### 2.7 Detection model construction

To explore the feasibility of using machine learning and deep learning algorithms to establish detection and discrimination models for EC, SA and ST on mutton using SWIR-HSI, the 1D-CNN network structures suitable for the discrimination of pathogenic bacteria contamination status and species were established in this study, and the detection model was established. At the same time, the typical linear modeling method partial least squares-discriminant analysis (PLS-DA) and the nonlinear modeling method SVM were also used to establish the corresponding models, and the different models were compared.

#### 2.7.1 One dimensional convolutional neural network

**Figure 2:**
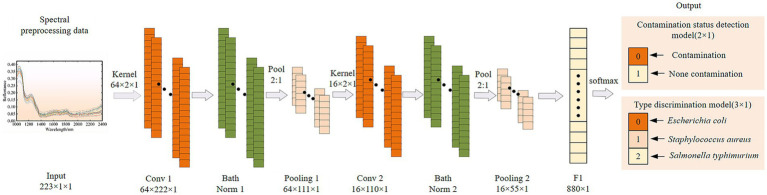
Structure diagram of a one dimension convolutional neural network (1D-CNN).

The CNN network structure built in this paper is shown in Figure 2, which is mainly composed of two one-dimensional (1D) convolutional layers, two maximum pooling layers and two fully connected (FC) layers. The spectrum of each sample contained 223 wavelengths, which were converted into a 1× 223 vector. After batch normalization, it is used as the input of 1D-CNN convolutional layer. The convolution kernel size and step size were set to 2 and 1, respectively. It is very important to learn model by properly configuring the number of convolution kernels (34). The number of kernels in the two convolutional layers was set to 128, 64, 32 and 16, respectively, and they were combined for model training. The optimal number of convolution kernels in each convolutional layer was determined according to the optimization results of model evaluation indicators. The CNN models usually add activation functions to the excitation layer to provide nonlinear modeling capabilities of the network. The commonly used activation functions are sigmoid, hyperbolic tangent function (tanh) and rectified linear unit (relu). They were used to establish CNN models, and the appropriate activation function was selected according to the model results. Adding a batch normalization layer between the convolutional and maximum pooling layers effectively avoids over-fitting and makes the debugging of hyperparameters easier. The deep features after the last pooling layer are flattened into one-dimensional vectors and then transmitted to the fully connected layer to establish the classification relationship between their features and the contamination status or species of pathogenic bacteria by the Softmax function. All samples were classified based on their probability for each category. To ensure fairness of model comparison, all 1D-CNN model training parameters were kept consistent. The batch size was set to 100, the maximum number of iterations was 200, the initial learning rate was 0.001, the learning rate decline was selected as ‘piecewise’, the learning rate decline factor is 0.1, and the ‘LearnRateDropPeriod’ was 200.

#### 2.7.2 Partial least squares-discriminant analysis

PLS-DA mainly performs multivariate statistical analysis of the data. In PLS-DA analysis, the explanatory data sets X and Y of the two data matrices are concatenated together for multi-source classification. In this study, the PLS-DA can not only de-compose the spectral data matrix, but also enhance the decomposition of the spectral data matrix by using pathogen contamination status or species labels to increase the spectral differences of different categories and improve the classification ability of the model (35). Internal cross-validation of PLS-DA was performed using leave-one-out and 10 fold cross-over methods.

#### 2.7.3 Support vector machine

As a supervised learning model, SVM can map the sample space into a high-dimensional feature space, which enables it to be transformed into linearly separable problems in the feature space (36). When SVM was used to establish the model of contamination status and species of pathogenic bacteria, the Radial Basis Function (RBF) was selected as the kernel function, and the optimal parameter combination of kernel function parameter (g) and penalty factor parameter (c) was sought by GA optimization algorithm.

### 2.8 Model evaluation and data processing environment

The accuracy was used to evaluate the performance of the model. Accuracy is the ratio of the number of correctly classified samples to the total number of samples. Higher accuracy indicates better model performance. The server environment used for model training is as follows: The processor is Intel(R) Core(TM)i7-10700F CPU @2.90GHz 2.90GHz, memory 16 GB, GPU graphics card is NVIDIA GeForce RTX 2060, operating system is 64-bit Windows10, The programming software was MatlabR2023a.

## 3 Results and discussion

### 3.1 Analysis of raw spectral data

**Figure 3:**
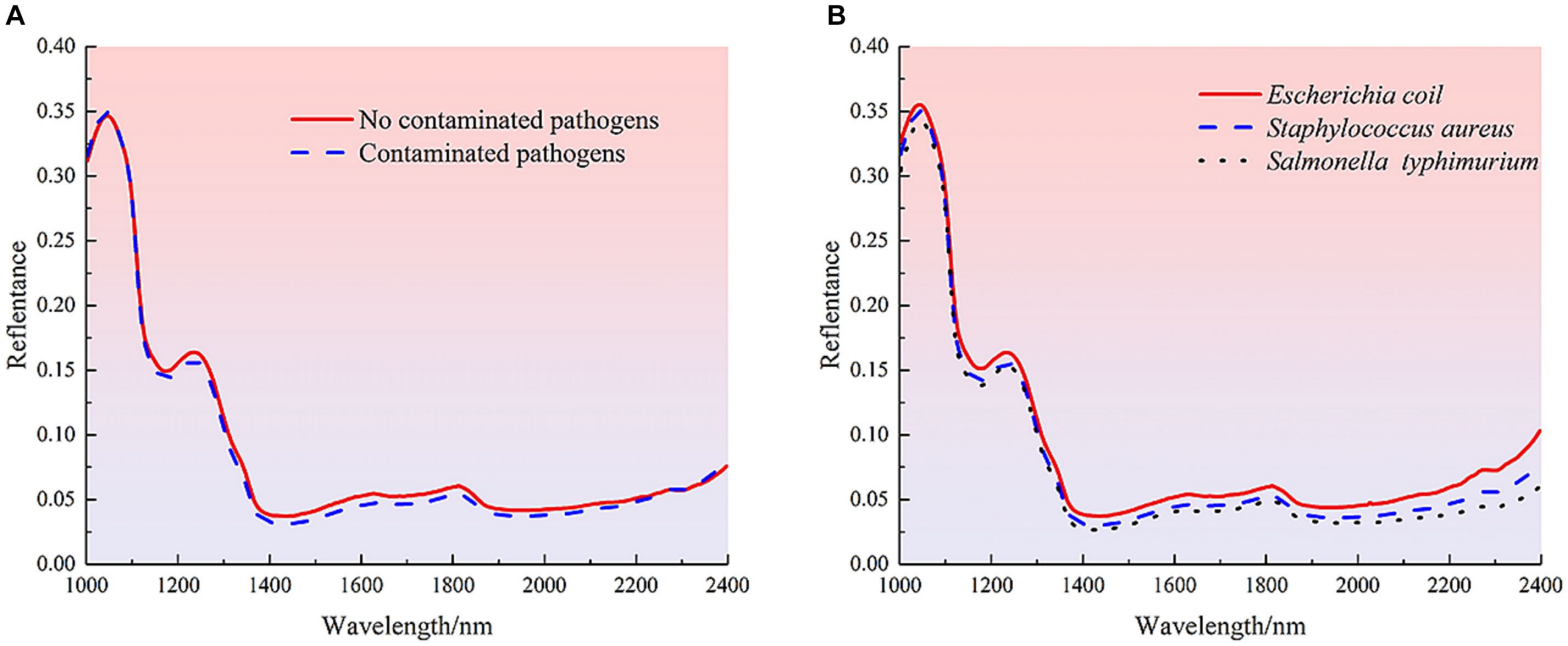
Spectral curves of different samples. **(A)** Average spectra of uncontaminated and contaminated pathogenic mutton samples. **(B)** Average spectra of meat samples contaminated with different species of pathogenic bacteria.

Near-infrared spectroscopy (NIR) is used to detect the internal quality information of samples by the consistent frequency doubling and frequency joining absorption caused by the non-resonant vibration of hydrogen-containing groups (C-H, O-H and N-H) in the substance molecules (37). The average spectral reflectance curves of 210 uncontaminated mutton samples and contaminated mutton samples with different species of bacteria are shown in Figure 3. The spectral curves of uncontaminated and contaminated mutton samples showed the same trend, and the average spectral curves of samples showed the similar trend. There were significant absorption peaks at 1040, 1235, 1813 and 2,272 nm wavelengths, but there were some differences in spectral reflectance. Among them, the absorption peak at 1040 nm was closely related to the stretching vibration of the N-H bond in the molecular structure of protein, and the ab-sorption peaks at 1235 nm and 1813 nm were related to the secondary and primary frequency of the C-H bond in the molecular structure of meat organic components, respectively. For the spectral absorption region of 2000–2,500 nm, the large spectral ab-sorption region is mainly caused by the combined frequency stretching vibration of the functional groups O-H, N-H and C-H, so this region shows a low spectral reflection value (38). For both uncontaminated and contaminated pathogenic samples, the mean reflectance of the uncontaminated pathogenic samples was higher than that of the contaminated samples. Samples contaminated with EC showed the highest reflectance, followed by SA, and ST showed the lowest. It is difficult to identify the pollution status and type only from the spectral reflectance. Therefore, it is necessary to combine ma-chine learning and deep learning algorithms to further establish detection models for effective detection.

### 3.2 Detection of pathogenic bacteria contamination status on mutton

The common foodborne pathogens on mutton are EC, SA and ST. The Chinese national standard has clear requirements for the contamination of pathogenic bacteria on fresh meat, among which ST and EC are required not to be detected, and SA is required to be controlled at 1000 CFU/g (2). Therefore, it is an important prerequisite to accurately distinguish whether mutton is contaminated with pathogenic bacteria. In this study, deep learning algorithms (1D-CNN) and machine learning algorithms (PLS-DA and SVM) were used to detect the contamination status of pathogenic bacteria in mutton based on the spectral information of 1,000–2,500 nm.

#### 3.2.1 Optimization and determination of hyperparameters of 1D-CNN model structure

To establish a 1D-CNN model suitable for the detection of pathogenic bacteria contamination in mutton, the original spectra were used to establish a classification model, and the effects of convolution kernel number and activation function on the CNN model were investigated. The appropriate number of convolution kernels was first determined according to the accuracy of the external validation set. When the accuracy of the external validation sets of the two models was the same, the appropriate number of convolution kernels was determined according to the accuracy of the test set. The appropriate number of convolution kernels enables the model to make full use of local and global features when training samples (39). The activation function was set as relu, and the optimal convolution kernel number parameter was selected. The results of pathogen contamination status detection based on full-band spectral data under different convolution kernel numbers are shown in Table 1.

**Table 1:** 1D-CNN models for pathogenic bacteria contamination status on mutton based on different numbers of convolution kernels.

Number of convolution kernels	Accuracy		
	Training set	Test set	External validation set
(128,64)	99.66%	86.90%	97.62%
(128,32)	99.67%	88.10%	95.24%
(128,16)	98.64%	83.33%	92.86%
(64,32)	99.66%	88.10%	97.62%
(64,16)	100.00%	90.48%	97.62%
(32,16)	99.66%	90.48%	95.24%

Table 1 shows that different numbers of convolution kernels have an obvious im-pact on the results of the pathogen contamination status detection model. When the number of first convolutional layers is 64, the results of the external validation set outperform the models with the number of convolution kernels being 32 and 128. When the second layer convolution kernel is 16 and 32, the results of the external validation set are the same, but the test set with the number of 16 is better than the model with the number of 32. Therefore, when the number of convolution kernels was (64, 16), the performance of 1D-CNN in detecting the contamination status of pathogenic bacteria was the best, with the accuracy of 97.62% for the external validation set and 90.48% for the test set, respectively. According to the reports of previous studies, different activation functions can also affect the model. When the convolution kernel of the 1D-CNN model is set to (64, 16), the effects of three activation functions, sigmoid, tanh and relu, on the detection model of pathogenic bacteria contamination status were discussed, and the results were shown in Table 2.

**Table 2:** 1D-CNN models for pathogenic bacteria contamination status on mutton based on different activation functions.

Activation function	Accuracy		
	Training set	Test set	External validation set
Relu	100.00%	90.48%	97.62%
Tanh	100.00%	92.86%	97.62%
Sigmoid	94.90%	85.71%	95.24%

As shown in Table 2, when using the1D-CNN models with three different activation functions to establish the detection models of pathogenic bacteria contamination status on mutton, the accuracy of all datasets was greater than 85%, indicating that three activation functions can be used to construct the 1D-CNN models for the detection of pathogenic bacteria contamination status on mutton. However, when the activation function is sigmoid, the performance of the model is significantly worse than that of tanh or relu. In addition, the accuracy of the test set and the external validation set of the sigmoid model was quite different, indicating that the stability of the model was not good. The results of training set and external validation set of tanh and relu models were the same, while the accuracy of test set of tanh model was higher than that of relu model. Therefore, tanh activation function is more suitable for establishing the detection model of pathogenic bacteria contamination in mutton. In the follow-up study, the number of convolution kernels in the pathogen contamination status detection model built by 1D-CNN was all (64, 16), and the activation function was tanh.

#### 3.2.2 Influence of different spectral data preprocessing on the model

The methods of 1D, 2D, MC and MSC were used to preprocess full-band spectral data. The PLS-DA, SVM and 1D-CNN models for the detection of pathogenic bacteria contamination on mutton were established using the preprocessed spectral data, and the effects of spectral preprocessing on different models were discussed. The results are shown in Table 3.

**Table 3:** Results of the detection model for pathogenic bacteria contamination status on mutton established by spectral data after different preprocessing method.

Model	Pretreatment method	Accuracy		
		Training set	Test set	External validation set
1D-CNN	None	100.00%	92.86%	97.62%
	1D	99.98%	85.71%	95.24%
	2D	100.00%	89.26%	95.24%
	MC	97.96%	90.48%	95.24%
	MSC	99.66%	86.90%	90.48%
PLS-DA	None	92.18%	90.14%	89.29%
	1D	89.12%	85.71%	83.33%
	2D	92.85%	90.48%	89.29%
	MC	86.73%	83.33%	79.76%
	MSC	87.07%	85.37%	83.33%
SVM	None	93.20%	86.39%	79.76%
	1D	98.30%	88.10%	88.76%
	2D	99.32%	91.67%	90.14%
	MC	83.33%	77.38%	74.83%
	MSC	96.94%	78.57%	80.95%

Table 3 shows that for PLS-DA and SVM models, the models established by the 2D preprocessed spectra are better than that of the original spectra. Compared with the model without preprocessing, the accuracy of test set of PLS-DA model was improved by 0.34%. The accuracy of the test set and the external validation set of the SVM model was increased by 5.28 and 10.38%, respectively, which indicated that 2D processing can significantly improve the nonlinear relationship between spectral data and contamination conditions. For the 1D-CNN model, the model without preprocessing had the best effect, and the accuracy of the model decreased after preprocessing. The results showed that the 1D-CNN model was easier to mine the spectral features related to contamination status from the original spectral data. In conclusion, 2D preprocessing could effectively achieve baseline correction of spectral data and remove background interference to enhance the detection performance of PLS-DA and SVM models. The spectral data used for subsequent feature extraction were preprocessed spectra by the 2D method.

#### 3.2.3 Extraction of spectral characteristic wavelengths

**Figure 4:**
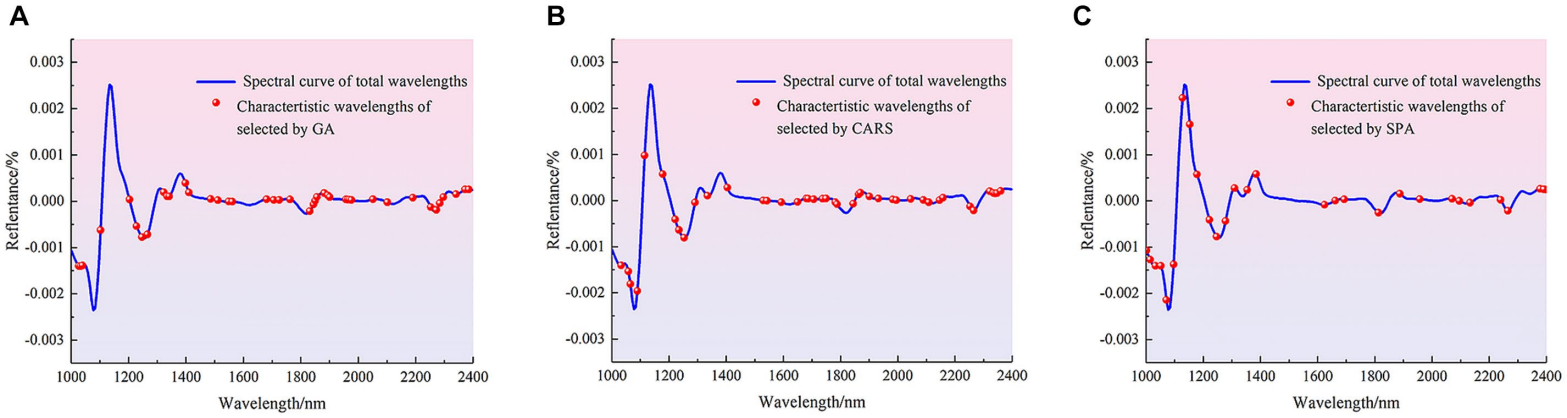
Distribution of characteristic wavelengths extracted by different methods in the full band. **(A)** GA. **(B)** CARS. **(C)** SPA.

The methods of CARS, GA and SPA were used to extract characteristic wave-lengths related to the contamination status of pathogenic bacteria on mutton. Among them, the main parameters of GA for feature wavelength extracting are set as follows: the initial population is 64, the mutation probability is 0.005, the genetic iteration number is 100, and the convergence rate is 0.5. The distribution of the characteristic wavelengths extracted using different methods in the full band is shown in Figure 4.

The number of characteristic wavelengths extracted using CARS, GA and SPA was 33, 35 and 15, respectively. The characteristic wavelengths extracted by GA were scattered in the range of 1,000–1,600 nm, and were mainly densely distributed in the range of 1,687–1810 nm, 1881–2007 nm and 2,152–2,400 nm. The characteristic wavelengths extracted using CARS were also distributed in the 1,071–1,253 nm and 1875–2,328 nm ranges, as well as in the vicinity of 1,000, 1,020, 1,543, 1,586, 1,693 and 1712 nm. The characteristic wavelengths extracted by SPA were mainly distributed at 1008–1385 nm and 2,196, 2,252 and 2,284 nm. Although there are differences in the distribution of spectral wavelength variables obtained from screening by different methods, these bands contribute significantly to the identification model for the presence or absence of pathogenic bacteria in mutton. The main reason was that when mutton was contaminated by pathogenic bacteria, the spectral curves obtained by using HSI were the spectra under the joint action of pathogenic bacteria and mutton. The bands with large differences in reflectance between the spectral curves of pure mutton and mutton contaminated by pathogenic bacteria were selected as the characteristic bands, and these bands are mainly related to the intensity change of functional group, including O-H, N-H and C-H, caused by pathogenic bacteria (40, 41). Different characteristic wavelengths were used to establish simplified models for the detection of pathogenic bacteria contamination in mutton, and the effects of characteristic wavelength extraction on PLS-DA, SVM and 1D-CNN models were discussed. The results are shown in Table 4.

**Table 4:** Results of detection models for pathogenic bacteria contamination status on mutton established by characteristic wavelengths extracted by different methods.

Model	Method of feature band screening	Number of bands	Accuracy		
			Training set	Test set	External validation set
1D-CNN	CARS	33	96.94%	85.71%	92.86%
	GA	35	98.64%	84.52%	95.24%
	SPA	15	94.56%	75.00%	95.24%
PLS-DA	CARS	33	86.73%	82.14%	83.33%
	GA	35	89.12%	86.90%	92.86%
	SPA	15	90.14%	82.14%	92.86%
SVM	CARS	33	98.98%	90.48%	90.48%
	GA	35	94.22%	89.29%	95.24%
	SPA	15	93.57%	86.90%	92.86%

Table 4 shows that for PLS-DA, SVM and 1D-CNN models, the models constructed by the characteristic wavelengths extracted by GA have the better effect. The results showed that GA was more effective than CARS and SPA in extracting the key spectral features related to contamination status of pathogenic bacteria on mutton. Compared with the model established by full band, the accuracy of the training set and test set of GA-PLS-DA and GA-SVM model decreased, while the accuracy of the external validation set increased. This could be attributed to the fact that GA may lose a small amount of useful information when extracting spectral features of uncontaminated and mutton samples contaminated with pathogenic bacteria. Compared with the 1D-CNN model the Tanh activation function established by full band (Table 2), the training set, test set and external validation set of the 1D-CNN model established by characteristic wavelengths were reduced by 1.36, 8.34 and 2.38%, respectively.

#### 3.2.4 Model comparison and optimal model selection

The methods of PLS-DA, SVM and 1D-CNN were used to establish the detection models of pathogenic bacteria contamination on mutton. For the models established by the full band spectra, the optimal model was the 1D-CNN model established by the original spectra, and its training set, test set and external validation set were 100.00, 92.86 and 97.62%, respectively (Table 3). For PLS-DA and SVM models, the effects of the models established after 2D preprocessing were all the best models, and the performance of SVM was better than that of PLS-DA. For the simplified models built by the characteristic wavelengths, the models built by the 35 characteristic wavelengths extracted by GA were all the optimal models. According to the comprehensive results of the three datasets, the optimal model was GA-SVM. The training set, test set and external validation set of the optimal simplified model GA-SVM were 94.22, 89.29 and 95.24%, respectively (Table 4). Compared with the 1D-CNN model established by the full band spectra, its training set, test set and external validation set were reduced by 5.78, 3.47 and 2.38%, respectively. In conclusion, the optimal model for the detection of pathogenic bacteria contamination on mutton is the 1D-CNN model established by the original spectra. Due to the powerful ability to extract feature and handle both linear and nonlinear relationships of 1D-CNN model, it showed a significant detection advantage over PLS-DA and SVM models. At the same time, the original band was used to establish the detection model, which omitted the preprocessing and feature extraction process, and the detection efficiency was improved. This conclusion was similar to previous studies (42–44). In addition, the simplified model established by using the characteristic bands reduced the complexity of the characteristics. Although the accuracy was slightly lower than that of the full-band model, the characteristic bands could provide some reference when developing a low-cost multispectral detector. Low-cost multispectral detectors were more suitable for real-time and rapid detection of large quantities of samples.

### 3.3 Establishment of the discrimination models of pathogenic bacteria species contaminated on mutton

After the detection of the contamination status of pathogenic bacteria on mutton, the discrimination of the species of pathogenic bacteria is particularly important. The limit requirements for different types of pathogenic bacteria on mutton are different. Rapid and accurate discrimination of pathogenic bacteria can effectively realize the safety detection of pathogenic bacteria. In this study, PLS-DA, SVM and 1D-CNN were used to establish the discrimination models of pathogenic bacteria species contaminated in mutton.

#### 3.3.1 Optimization and determination of hyperparameters of 1D-CNN model structure

In order to establish a 1D-CNN model for the discrimination of pathogenic bacteria contaminated in mutton, the original spectra were used to construct a classification model, and the effects of convolution kernel number and activation function on the CNN model were investigated. The activation function was set to relu, and the different convolution kernel numbers in Table 5 were used to establish the pathogen species discrimination model.

**Table 5:** 1D-CNN models for pathogenic bacteria species discrimination based on different numbers of convolution kernels.

Number of convolution kernels	Accuracy		
	Training set	Test set	External validation set
(128,64)	100.00%	100.00%	100.00%
(128,32)	100.00%	100.00%	100.00%
(128,16)	100.00%	100.00%	100.00%
(64,32)	100.00%	100.00%	100.00%
(64,16)	100.00%	100.00%	100.00%
(32,16)	100.00%	100.00%	100.00%

The results showed that the number of convolution kernels had no significant effect on the results of the pathogen species discrimination model. The accuracy of all models was 100.00%. The results showed that the 1D-CNN model was very suitable for establishing the discrimination model of pathogenic bacteria in mutton. According to the fact that the smaller the convolution sum, the smaller the computational cost of the model, the 1D-CNN with convolution sum of (32, 16) was selected to establish the pathogen species discrimination model.

When the convolution kernel of the 1D-CNN model was set to (32, 16), the effects of three activation functions (sigmoid, tanh and relu) on the pathogen species dis-crimination model were investigated. The results showed that all three activation functions could be used to establish the pathogen species discrimination 1D-CNN model, and the type of activation function had little effect on the model (Table 6). When the activation functions were relu or sigmoid, the accuracy of the test set of the model was 97.62%, and the accuracy of other data sets was 100.00%. Therefore, tanh activation function was selected to establish the discriminant model of pathogenic bacteria in mutton. In the subsequent study, the number of convolution kernels in the pathogen species discrimination model established by 1D-CNN was (32, 16), and the activation function was tanh.

**Table 6:** 1D-CNN model for pathogenic bacteria species discrimination based on different activation functions.

Activation function	Accuracy		
	Training set	Test set	External validation set
Relu	100.00%	97.62%	100.00%
Tanh	100.00%	100.00%	100.00%
Sigmoid	100.00%	97.62%	100.00%

#### 3.3.2 Effects of spectral preprocessing on different models

Different pre-processed spectral data were used to establish discrimination models of pathogenic bacteria species contaminated in mutton, and the effects of different processing methods on PLS-DA, SVM and 1D-CNN models were discussed. The results are shown in Table 7.

**Table 7:** Results of discrimination models for pathogenic bacteria species based on the spectra with different pretreatment methods.

Model	Pretreatment method	Accuracy		
		Training set	Test set	External validation set
1D-CNN	None	100.00%	100.00%	100.00%
	1D	100.00%	100.00%	100.00%
	2D	100.00%	100.00%	100.00%
	MC	100.00%	100.00%	100.00%
	MSC	100.00%	100.00%	100.00%
PLS-DA	None	99.32%	97.62%	100.00%
	1D	98.64%	100.00%	100.00%
	2D	99.32%	100.00%	100.00%
	MC	98.64%	97.62%	100.00%
	MSC	99.32%	97.62%	100.00%
SVM	None	88.44%	80.95%	85.71%
	1D	100.00%	92.86%	100.00%
	2D	100.00%	100.00%	100.00%
	MC	88.44%	85.71%	80.95%
	MSC	98.64%	85.71%	80.95%

Table 7 shows that for the PLS-DA model, the model with 2D preprocessing has the best effect, and the model performance is slightly improved compared with no pre-treatment. For the SVM model, the performance of the 2D pre-processing model was significantly improved. Compared with the model based on the original spectra, the accrary of the training set, test set and external validation set was improved by 11.56, 19.05 and 14.29%, respectively. The results showed that 2D preprocessing effectively strengthened the nonlinear relationship between spectral data and pathogenic bacteria species. For the 1D-CNN model, the effects of all models were 100.00%, indicating that preprocessing had no effect on the 1D-CNN model. The above studies indicated that spectral preprocessing could improve the performance of PLS-DA and SVM models (45), but had no effect on the 1D-CNN models, when building species discrimination models of pathogenic bacteria in mutton.

#### 3.3.3 Extraction of spectral characteristic wavelengths

**Figure 5:**
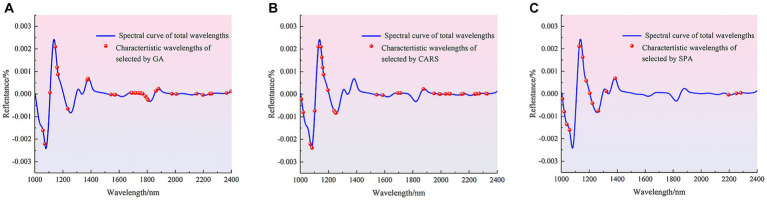
Distribution of characteristic wavelengths extracted by different methods. **(A)** GA. **(B)** CARS. **(C)** SPA.

The methods of GA, CARA and SPA were used to extract the characteristic wave-lengths associated with pathogenic bacteria species in mutton. The distribution of characteristic wavelengths extracted using different methods in the full band is shown in Figure 5.

Figure 5 shows that that the characteristic wavelengths screened by GA mainly include 1,030, 1,102, 1,340, 1,680, 1705, 1724, 1762, 1960 and 1976 nm, and the distribution of characteristic bands is relatively dense in the range of 1,203–1,266, 1,397–1,561 and 1831–1900 nm. The characteristic wavelengths screened by CARS were mainly distributed in the range of 1,033–1,064 and 1,649–2,158 nm, and also distributed around 1,115, 1,178, 1,222–1,335, 1,404, 1,529, 1,542, 1,592, 2,350 nm. The characteristic wavelengths screened by SPA were mainly distributed in the vicinity of 1,310, 1,385, 2,240, 1820 and 2,390 nm, and also distributed in the range of 1,002–1,052, which contained most of the peaks and trough after the second derivative treatment. The characteristic bands mainly related to the difference between different pathogenic bacteria. Then the characteristic bands extracted by different methods were used to establish the pathogen species discrimination model, as shown in Table 8.

**Table 8:** Results of discrimination models for pathogenic bacteria species established with characteristic wavelengths extracted by different methods.

Model	Method of feature band screening	Number of bands	Accuracy		
			Training set	Test set	External validation set
1D-CNN	CARS	42	100.00%	95.24%	100.00%
	GA	42	100.00%	97.61%	100.00%
	SPA	29	100.00%	92.86%	100.00%
PLS-DA	CARS	42	100.00%	100.00%	100.00%
	GA	42	100.00%	100.00%	100.00%
	SPA	29	100.00%	100.00%	100.00%
SVM	CARS	42	100.00%	100.00%	100.00%
	GA	42	100.00%	100.00%	100.00%
	SPA	29	100.00%	100.00%	100.00%

As shown in Table 8, the all datasets accuracy of PLS-DA and SVM models con-structed with characteristic wavelengths extracted by GA, CARS and SPA for discriminating the species of pathogenic bacteria on mutton was 100.00%. It shows that all of three methods could effectively extract the key spectral features of different types of pathogenic bacteria. Compared with the full-band model, the accuracy of PLS-DA and SVM was improved. Because the number of characteristic wavelengths extracted by SPA was smallest, the PLS-DA and SVM models established by the characteristic wave-lengths extracted by SPA were faster. Therefore, SPA combined with machine learning can be effectively used to establish simplified models for the species discrimination of pathogenic bacteria contaminated in mutton. On the contrary, for the 1D-CNN model, the accuracy of the models based on the characteristic wavelengths extracted by different methods was lower than that of the full-band model, indicating that the feature extraction lost some features available (44).

#### 3.3.4 Comparison of model preferences

The methods of PLS-DA, SVM and 1D-CNN were used to establish the species discrimination models of pathogenic bacteria in mutton. For the full-band model, the accuracy of all datasets of 2D-SVM and all 1D-CNN models was 100.00%, and the model effect was satisfactory. For the model built by the characteristic wavelengths, the PLS-DA and SVM models based on the characteristic band had good effects, and the accuracy of all datasets was 100.00%. However, the accuracy of the 1D-CNN model based on the characteristic wavelengths was lower than that of the full band. The results showed that for the machine learning model, preprocessing could improve model accuracy, and feature extraction could effectively remove the features that were not related to the pathogen species in the full-band spectral information. Although the model based on the characteristic wavelengths had a good effect, and the simplified model could provide a basis for the development of multispectral detection instruments, it needed to go through the process of manual preprocessing and feature extraction, which increased the complexity of establishing species discrimination models of pathogenic bacteria in mutton using SWIR-HSI. When the 1D-CNN model was used, the effect of the model based on the original spectrum was optimal, and its end-to-end detection mode effectively improved the efficiency of model detection. In conclusion, considering the computational complexity of features and models, the 1D-CNN model based on the original spectra was the optimal model when the SWIR-HSI was used for the discrimination the species of pathogenic bacteria in mutton.

At present, the traditional culture method is the most common and reliable means of pathogenic bacteria detection, but it needs professional operators and strict testing environment, and the detection time is long and the efficiency is low. Compared with the traditional culture detection of pathogenic bacteria, HSI can be more quickly used to rough select the contamination status of pathogenic bacteria in mutton and discrimination their types. However, in the early stage of the research, due to the small amount of data, the generalization performance of the built model was not strong. In the follow-up study, we should consider the influence of multiple factors on the model, such as the presence of indigenous microbiota, breeds and parts of mutton, etc., to enhance the generalization performance of the model, so that the method proposed in this study was more feasible.

## 4 Conclusion

The feasibility of using SWIR-HSI (1000–2,500 nm) combined with traditional machine learning and deep learning methods to detect the pathogenic bacteria contamination on mutton was explored in this study. The 1D-CNN model which was suitable for detection the contamination status and species of pathogenic bacteria in mutton was constructed and optimized according to the full-band spectra. The effects of different preprocessing methods and characteristic wavelengths extraction on different models are explored. The results showed that the number of convolution kernels and the type of activation function in the 1D-CNN model could significantly affect the detection model for contamination status of pathogenic bacteria on mutton, but had little effect on the discrimination model of the contaminated pathogenic bacteria on mutton. For the detection of pathogenic bacteria contamination status on mutton, 2D preprocessing could improve the accuracy of PLS-DA and SVM, but it had no effect on the 1D-CNN model. Using GA method to extract characteristic wavelengths could simplify the input of the model and reduce the complexity of the model. The overall results showed that the 1D-CNN model based on the original spectra was the best model for the detection of pathogenic bacteria contamination status on mutton. For the species discrimination of pathogenic bacteria contaminated on mutton, the spectral preprocessing affected the model in the same way as the contamination detection model. The 2D preprocessing method could effectively improve the accuracy of PLS-DA and SVM models, while it had no effect on 1D-CNN model. When GA, CARS and SPA were used to extract the characteristic wavelengths to establish the simplified model, the performance of the 1D-CNN model was reduced compared with that of the full-band model. But the effects of PLS-DA and SVM models based on characteristic wavelengths were satisfactory. In conclusion, the combination of SWIR-HSI with traditional machine learning and deep learning methods could effectively detect the contamination status and identify the species of pathogenic bacteria in mutton, and the performance of deep learning model was better than that of machine learning. The results provide theoretical basis and technical support for the effective realization of rapid nondestructive detecting of pathogenic bacteria. For future research, the detection limit of GB29921-2013 for different pathogenic bacteria will be considered too quickly distinguish their concentration levels, and the detection limit of SWIR-HSI for pathogenic bacteria detection will be discussed.

## Data availability statement

The raw data supporting the conclusions of this article will be made available by the authors, without undue reservation.

## Author contributions

ZB: Conceptualization, Data curation, Formal analysis, Investigation, Methodology, Software, Supervision, Visualization, Writing – original draft. DD: Data curation, Investigation, Methodology, Supervision, Writing – review & editing. RZ: Conceptualization, Funding acquisition, Project administration, Resources, Writing – review & editing. FX: Data curation, Software, Validation, Visualization, Writing – review & editing. CY: Data curation, Writing – review & editing. JY: Data curation, Writing – review & editing. YZ: Data curation, Validation, Writing – review & editing. LK: Funding acquisition, Resources, Writing – review & editing.
